# Rosanoid diterpenoids: structural diversity, classification and biological activities

**DOI:** 10.5599/admet.3165

**Published:** 2026-03-06

**Authors:** Sabrin R. M. Ibrahim, Hani Z. Asfour, Gamal A. Mohamed, Nabil A. Alhakamy, Hossam M. Abdallah, Hagar M. Mohamed

**Affiliations:** 1 Preparatory Year Program, Department of Chemistry, Batterjee Medical College, Jeddah 21442, Saudi Arabia; 2 Department of Pharmacognosy, Faculty of Pharmacy, Assiut University, Assiut 71526, Egypt; 3 Department of Clinical Microbiology and Immunology, Faculty of Medicine, King Abdulaziz University, Jeddah 21589, Saudi Arabia; 4 Department of Natural Products and Alternative Medicine, Faculty of Pharmacy, King Abdulaziz University, Jeddah 21589, Saudi Arabia; 5 Department of Pharmaceutics, Faculty of Pharmacy, King Abdulaziz University, Jeddah 21589, Saudi Arabia; 6 Mohamed Saeed Tamer Chair for Pharmaceutical Industries, King Abdulaziz University, Jeddah 21589, Saudi Arabia; 7 Department of Medical Laboratory Analysis, College of Medical & Health Sciences, Liwa University, Abu Dhabi 41009, United Arab of Emirates; 8 Department of Applied Medical Chemistry, Medical Research Institute, Alexandria University, Alexandria, Egypt

**Keywords:** ent-rosane diterpenoids, rosane diterpenoid, *Euphorbiaceae*, natural products, drug discovery, sustainable development

## Abstract

**Background and purpose:**

Rosanoid diterpenoids, including ent-rosane and rosane diterpenoids, are structurally unique and bioactive subclass diterpenes characterized by a tricyclic carbon skeleton. This work aims to provide a comprehensive review of the literature on these diterpenoids from 1975. to September 2025., including their occurrence, structural diversity, and biological activities.

**Approach:**

An extensive literature search was conducted through scientific databases (ScienceDirect, PubMed, Scopus, Web of Science, and Google Scholar) and publishers’ webpages (Elsevier, Wiley, ACS, RSC, Taylor & Francis, Springer, Bentham, Thieme, and MDPI), covering reports from 1975 to September 2025.

**Key Results:**

Rosanoid diterpenoids have been isolated from various natural sources, including fungi, liverworts, and higher plant families such as *Euphorbiaceae, Lamiaceae, Alismataceae, Asteraceae, Velloziaceae, and Celastraceae*. They are predominantly found in *Euphorbia* species, revealing their chemotaxonomic relevance to the *Euphorbiaceae* family. These compounds exhibit extensive structural diversity, encompassing a broad spectrum of biological activities, including anti-inflammatory, antimicrobial, antiviral, cytotoxic, enzyme-inhibitory, neuroactive, and anti-adipogenic effects.

**Conclusion:**

The reported findings highlight the chemical variability and pharmacological potential of rosanoid diterpenoids, making them promising building blocks for future drug discovery and natural product development. However, further studies are warranted to explore their pharmacokinetics, mechanisms of action, safety profiles, and biosynthetic pathways.

## Introduction

Sustainable healthcare practices that prioritize natural remedies and environmental stewardship provide an integrated approach to health by utilizing natural products. The use of natural products and their derived preparations is steadily growing in a variety of industries, including agriculture, food, cosmetics, and veterinary and human medicines [1]. Their growing significance arises from the fact that they primarily rely on renewable biological resources, which aligns with the principles of a sustainable and circular bioeconomy, reducing environmental impact while encouraging innovation in green chemistry [2,3].

For thousands of years, medicinal plants have been used as natural sources of treatment for various illnesses [4-6]. In addition, their bioactive constituents have been proven to have significant therapeutic applications and serve as lead molecules for drug discovery [7-9]. Terpenoids are among the vast and varied groups of natural metabolites derived from five-carbon isoprene building blocks through the condensation and subsequent modification of isoprene units in various ways, including cyclization and/or oxygenation [1,10]. These compounds have been reported from plants, animals, algae, fungi, coral, and other organisms, including different types such as kaurene, daphnane, abietane, tiglilane, pimarane, dolabellane, labdane, jatrophane, dolastane, casbane, tonantzitlolone, and miscellaneous [11,12]. They are crucial for the growth and development of plants. Terpenoids are not only essential for plant growth and ecological adaptation but also have been widely utilized in dietary supplements, illness prevention, and general human wellness [8,13].

**Figure 1.**
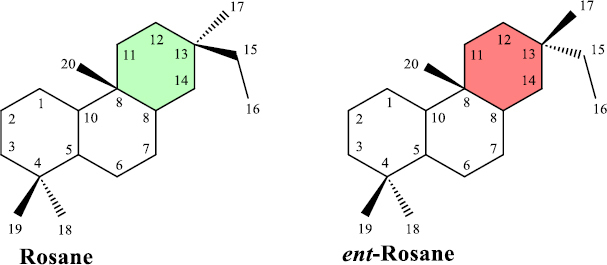
Basic skeletons of rosane and ent-rosane diterpenoids

Diterpenoids represent one of the largest classes of terpenoids, which are biosynthesized from four isoprene units via geranylgeranyl diphosphate (GGPP) [14]. Among them, rosanoid diterpenoids, including ent-rosane and rosane, are relatively uncommon subclasses of tricyclic compounds characterized by a 6/6/6 fused-ring system (Figure 1).

**Figure 2.**
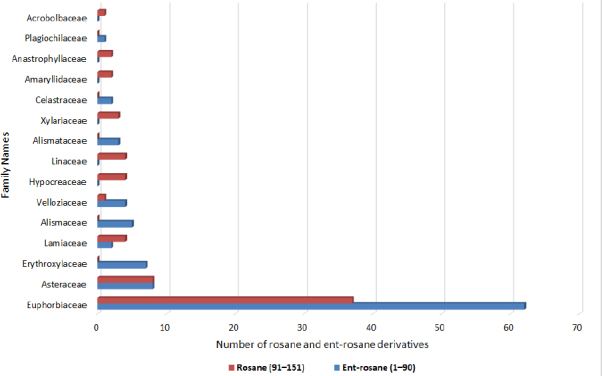
Distribution of rosanoid diterpenoids (ent-rosane and rosane) across different families based on reported literature from 1975 to 2025

Ent-rosane diterpenoids have been reported from various plant families, including *Euphorbiaceae*, *Lamiaceae*, *Alismataceae*, *Asteraceae*, *Velloziaceae*, and *Celastraceae*, with the genera *Euphorbia*, *Sagittaria*, *Vellozia*, *Trichogonia*, and *Maytenus* recognized as particularly rich sources (Figure 2).

**Figure 3.**
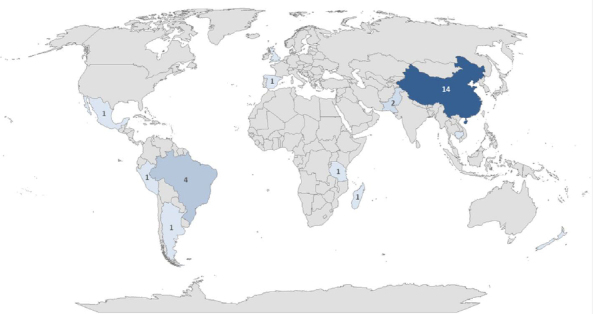
Geographical distribution of the number of rosanoid diterpenoid-producing species per country

This distribution indicates the chemotaxonomic significance and high-yielding taxa for further chemical and biological research. These plants are distributed across tropical and subtropical regions such as China, Brazil, Peru, Madagascar and Spain [15-18]. While rosane diterpenoids have been identified in liverworts, higher plants, and fungi (Figure 3).

These compounds exhibit structural diversity and a range of biological activities, including anti-inflammatory, antimicrobial, cytotoxic, antiviral, anti-osteoclastogenic, and antiadipogenic effects. Despite their structural diversity and pharmacological properties, no comprehensive review exists that examines their occurrence, structural variations, and biological relevance. Accordingly, this review provides a comprehensive overview of rosane-type diterpenoids, including their sources, structural classification, and biological activities. By integrating data reported up to 2025, this review aims to serve as a valuable reference for future studies on the chemistry and biological activities of this group of diterpenoids. This article also highlights the significant translation gaps, particularly in the areas of pharmacokinetics, metabolic stability, and toxicity and suggests integrating early ADMET/DMPK studies with bioactivity-based lead selection of rosanoid diterpenoid potential candidates.

## Search methodology

### Search strategy

An extensive literature search was conducted through scientific databases (ScienceDirect, PubMed, Scopus, Web of Science, and Google Scholar) and publishers’ webpages (Elsevier, Wiley, ACS, RSC, Taylor & Francis, Springer, Bentham, Thieme, and MDPI), focusing on rosanoid diterpenoids (rosane- and ent-rosane-type diterpenoids) reported in the literature up to September 2025. The search was done using the keywords: “Rosanoid diterpenoids”, “Rosane diterpenoids”, “ent-Rosane diterpenoids”, “Rosanoid diterpenoids + isolation”, “Rosanoid diterpenoids + structure elucidation”, “Rosanoid diterpenoids + biological activity”, “Rosanoid diterpenoids + pharmacology”, “Rosanoid diterpenoids + cytotoxicity”, “Rosanoid diterpenoids + anti-inflammatory”, “Rosanoid diterpenoids + antimicrobial”, “Rosanoid diterpenoids + anticancer”, and “Rosanoid diterpenoids + natural sources”.

### Inclusion and exclusion criteria

Published articles, reviews, and book chapters available in the above scientific databases that reported the isolation, structural elucidation, occurrence, and biological activities of natural rosanoid diterpenoids were included. However, the reported studies from non-peer-reviewed journals, irrelevant reports, and papers written in non-English without an English abstract were excluded.

## Classification of rosanoid diterpenoids

**Figure 4.**
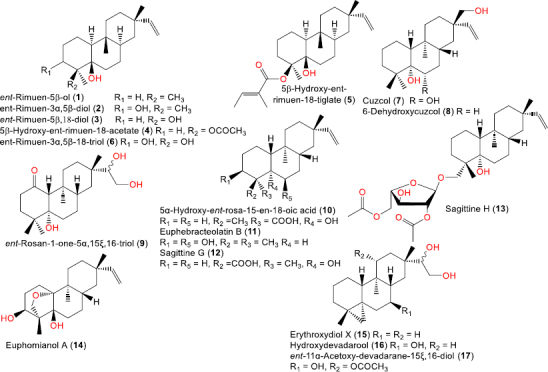
Chemical structures of saturated ent-rosane diterpenes (**1**-**17**).

**Figure 5.**
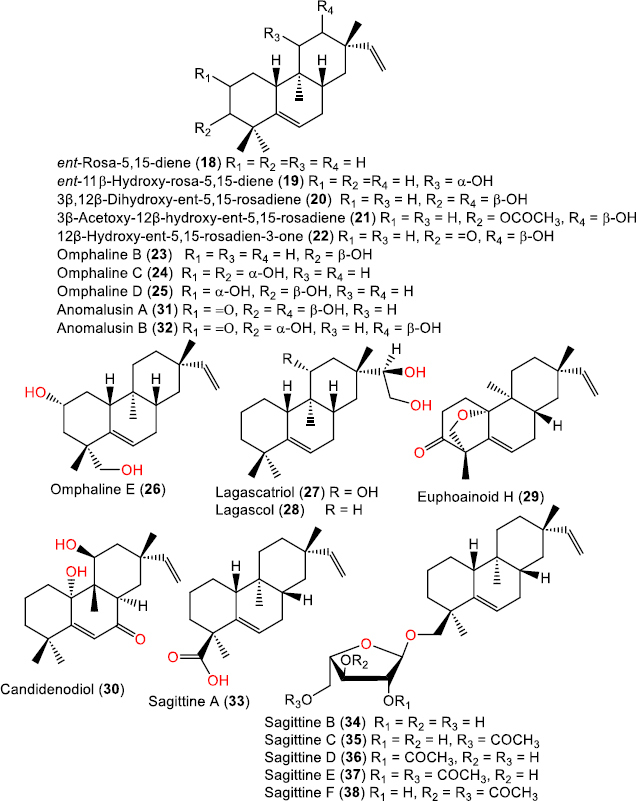
Chemical structures of ent-rosane diterpenoids with Δ^4^(^5^) unsaturation in rings A and B (**18-38**).

**Figure 6.**
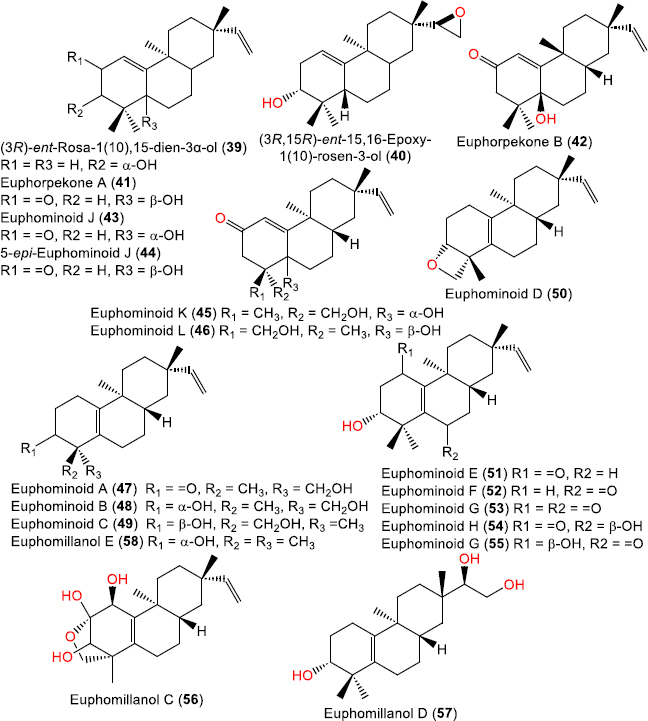
Chemical structures of Δ^1^(^10^)-unsaturated (**39-46**) and Δ5(^10^) and Δ^15^(^16^)-unsaturated ent-rosanes (**47**-**58**)

**Figure 7.**
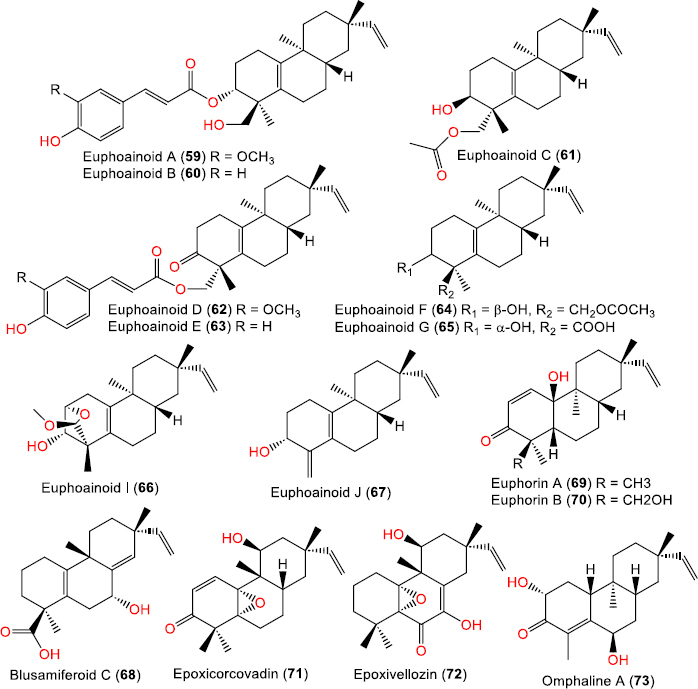
Chemical structures of Δ^5^(^10^), Δ^15^(^16^)-unsaturated (**59**-**67**) and other unsaturated ent-rosanes (**68**-**73**)

**Figure 8.**
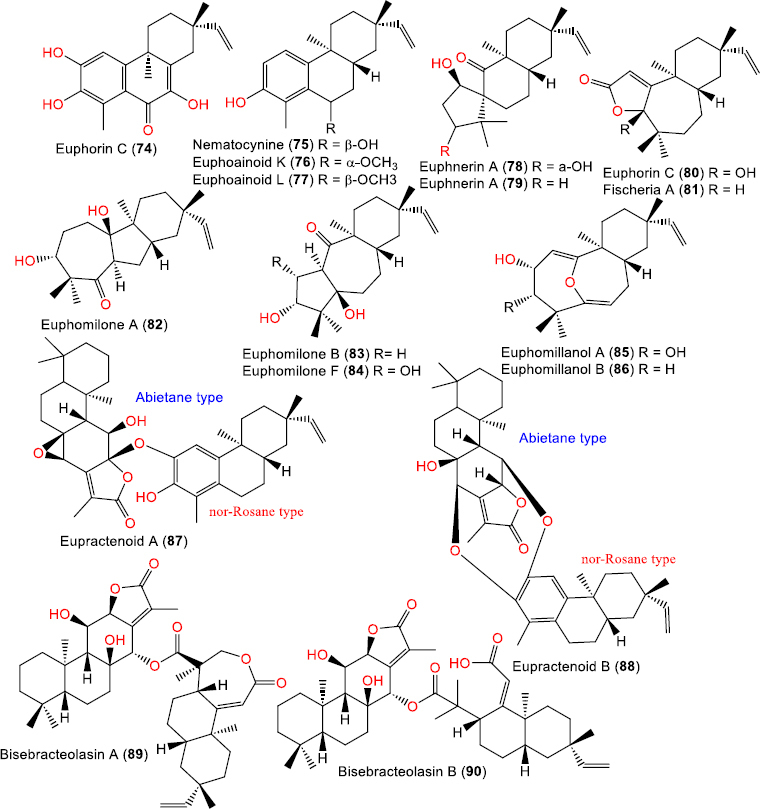
Chemical structures of aromatic (**74-77**) and modified skeletons (**78**-**86**), and dimeric ent-rosanes (**87-90**)

**Figure 9.**
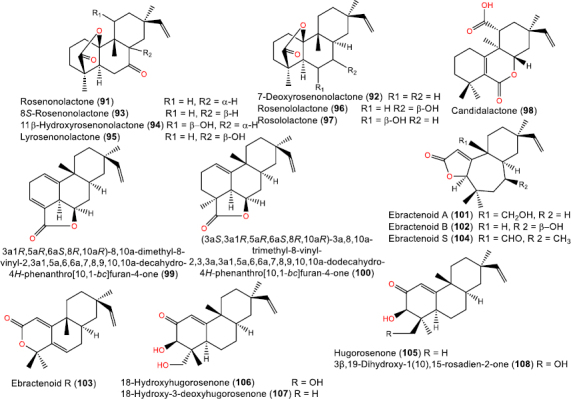
Chemical structures of lactone- (**91-104**) and ketone-containing (**105-108**) rosane derivatives

**Figure 10.**
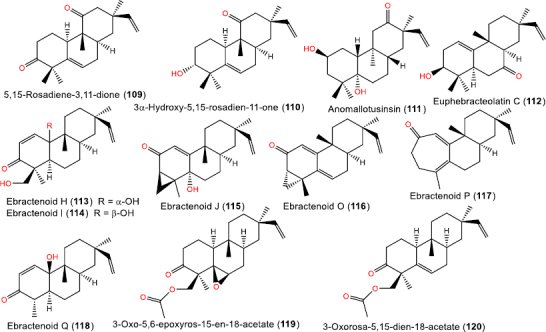
Chemical structures of ketone-containing (**109**-**120**) rosane diterpenoids

**Figure 11.**
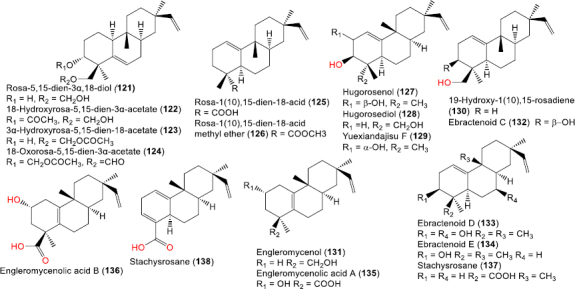
Chemical structures of rosadiene and ester derivatives (**121**-**138**)

**Figure 12.**
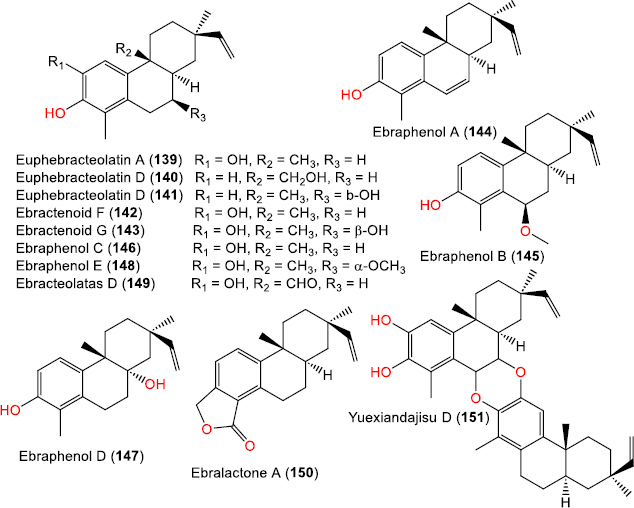
Chemical structures of aromatic (**139**-**150**) and dimeric (**151**) derivatives

Rosanoid diterpenoids are biosynthetically derived from geranylgeranyl diphosphate through an ent-copalyl diphosphate (ent-CPP) intermediate, followed by a series of cyclization, oxidation, and rearrangement reactions [19]. In general, rosanoid diterpenoids have a tricyclic C20 framework that undergoes substantial cyclization, accounting for the considerable structural variation documented in the literature. In the present review, the classification is mainly based on fundamental skeletal features that can be uniformly identified throughout reported studies, particularly (i) the number and position of the double bonds (unsaturation pattern), (ii) the extent and placement of oxygenation (*e.g.* hydroxyl, carbonyl, carboxyl, and ester substituents), and (iii) the presence of ring modifications such as lactone formation, aromatization, epoxidation, dimerization, and rearrangement/seco frameworks. Accordingly, the reported ent-rosane diterpenoids are categorized into major subclasses based on their unsaturation patterns within the tetracyclic skeleton, providing a practical framework for comparing related analogues (Table 1; Figures 4 to 8). In parallel, rosane diterpenoids are shown to be structurally diverse, including less common aromatic and dimeric derivatives, rosadiene/ester analogues, and lactone- and ketone-containing metabolites (Table 2; Figures 9 to 12). Overall, this classification goal is to present a logical structural map of rosanoid diterpenoids and to highlight the main modification patterns.

**Table 1.** List of reported ent-rosane diterpenoids (molecular weights, formulae, source and location)

Compound name/class	Molecular weight	Molecular formula	Plant, family, part used and location	Ref.
Saturated ent-rosane diterpenoids				
ent-Rimuen-5β-ol (**1**)	290	C_20_H_34_O	*Trichogonia salviaefolia* Gardner (*Asteraceae*), aerial parts, Bahia, Brazil,	[20]
			*Trichogonia villosa* Sch.Bip. exBaker (*Asteraceae*), aerial parts, Bahia, Brazil	
ent-Rimuen-5β,18-diol (**2**)	292	C_19_H_32_O_2_	*Trichogonia salviaefolia* Gardner (*Asteraceae*), aerial parts, Bahia, Brazil,	
			*Trichogonia villosa* Sch.Bip. ex Baker (*Asteraceae*), aerial parts, Bahia, Brazil	
ent-Rimuen-3α,5β-diol (**3**)	306	C_20_H_34_O_2_	*Trichogonia salviaefolia* Gardner (*Asteraceae*), aerial parts, Bahia, Brazil	
			*Trichogonia villosa* Sch.Bip. ex Baker (*Asteraceae*), aerial parts, Bahia, Brazil	
5β-Hydroxy-ent-rimuen-18-acetate (**4**)	334	C_21_H_34_O_3_	*Trichogonia villosa* Sch.Bip. ex Baker (*Asteraceae*), aerial parts, Bahia, Brazil	
5β-Hydroxy-ent-rimuen-18- tiglate (**5**)	374	C_24_H_38_O_3_	*Trichogonia salviaefolia* Gardner (*Asteraceae*), aerial parts, Bahia, Brazil	
ent-Rimuen-3α,5β-18-triol (**6**)	308	C_19_H_32_O_3_	*Trichogonia salviaefolia* Gardner (*Asteraceae*), aerial parts, Bahia, Brazil	
Cuzcol (**7**)	322	C_20_H_34_O_3_	*Maytenus cuzcoina Loesener*, (*Celastraceae*), root bark, Huayllabamba-Urquillos, Urubamba, Cusco (Perú)	[21]
6-Dehydroxycuzcol (**8**)	306	C_20_H_34_O_2_	*Maytenus cuzcoina Loesener*, (*Celastraceae*), root bark, Huayllabamba-Urquillos, Urubamba, Cusco (Perú)	[21]
ent-Rosan-1-one-5α,15ξ,16-triol (**9**)	338	C_20_H_34_O_4_	*Erythroxylum barbatum* O. E. Schulz (*Erythroxylaceae*), roots and trunk heartwood, Acarape, Ceará State, Northeast of Brazil	[22]
5α-Hydroxy-ent-rosa-15-en-18-oic acid (**10**)	320	C_20_H_32_O_3_	*Sagittaria pgymaea* Miq. (*Alismaceae*), herb, Nanning, Guangxi, China	[23]
Euphebracteolatin B (**11**)	306	C_20_H_34_O_2_	*Euphorbia ebracteolata* Hayata, (*Euphorbiaceae*), root, Changchun, Jilin, China	[24]
			*Euphorbia ebracteolata* Hayata, (*Euphorbiaceae*), root, Chuzhou, Anhui, China	[19]
Sagittine G (**12**)	320	C_20_H_32_O_3_	*Sagittaria sagittifolia L.*, (*Alismaceae*), herb, Nanning, Guangxi, China	[25]
			*Sagittaria trifolia* var. *sinensis* (*Sims*) Makino, (*Alismataceae*), herb, Nanning, Guangxi Zhuang Autonomous Region, China	[18]
Sagittine H (**13**)	522	C_29_H_46_O_8_	*Sagittaria trifolia* var. *sinensis* (*Sims*) Makino, (*Alismataceae*), herb, Nanning, Guangxi Zhuang Autonomous Region, China	[18]
Euphomianol A (**14**)	320	C_20_H_32_O_3_	*Euphorbia milii* Des Moul., (*Euphorbiaceae*), aerial parts, Baoshan, Yunnan, China	[26]
Erythroxydiol X (**15**)	306	C_20_H_34_O_2_	*Erythroxylum barbatum* O. E. Schulz (*Erythroxylaceae*), roots and trunk heartwood, Acarape, Ceará State, Northeast of Brazil	[22]
Hydroxydevadarool (**16**)	322	C_20_H_34_O_3_	*Erythroxylum barbatum* O. E. Schulz (*Erythroxylaceae*), roots and trunk heartwood, Acarape, Ceará State, Northeast of Brazil	[22]
ent-11α-Acetoxy-devadarane--15ξ,16-diol (**17**)	364	C_22_H_36_O_4_	*Erythroxylum barbatum* O. E. Schulz (*Erythroxylaceae*), roots and trunk heartwood, Acarape, Ceará State, Northeast of Brazil	[22]
*Ent*-rosane diterpenoids with Δ^4^(^5^) unsaturation in rings A and B				
ent-Rosa-5,15-diene (**18**)	272	C_20_H_32_	-	[27]
ent-11β-Hydroxy-rosa-5,15-diene (**19**)	288	C_20_H_32_O	-	[27]
3β,12β-Dihydroxy-ent-5,15-rosadiene (**20**)	304	C_20_H_32_O_2_	*Croton niveus* Jacq., (*Euphorbiaceae*), aerial parts, Melaque, State of Jalisco, México	[28]
3β-Acetoxy-12β-hydroxy-ent-5,15-rosadiene (**21**)	346	C_22_H_34_O_3_	*Croton niveus* Jacq., (*Euphorbiaceae*), aerial parts, Melaque, State of Jalisco, México	[28]
12β-Hydroxy-ent-5,15-rosadien-3-one (**22**)	302	C_20_H_30_O_2_	*Croton niveus* Jacq., (*Euphorbiaceae*), aerial parts, Melaque, State of Jalisco, México	[28]
Omphaline B (**23**)	288	C_20_H_32_O	of *Omphalea oppositifolia* (*Willd*.) L.J. Gillespie, (*Euphorbiaceae*), leaves and twigs, Moramanga, Alaotra-Mangoro, Madagascar	[29]
Omphaline C (**24**)	304	C_20_H_32_O_2_	of *Omphalea oppositifolia* (*Willd*.) L.J. Gillespie, (*Euphorbiaceae*), leaves and twigs, Moramanga, Alaotra-Mangoro, Madagascar	[29]
Omphaline D (**25**)	304	C_20_H_32_O_2_	of *Omphalea oppositifolia* (*Willd*.) L.J. Gillespie, (*Euphorbiaceae*), leaves and twigs, Moramanga, Alaotra-Mangoro, Madagascar	[29]
Omphaline E (**26**)	304	C_20_H_32_O_2_	of *Omphalea oppositifolia* (*Willd*.) L.J. Gillespie, (*Euphorbiaceae*), leaves and twigs, Moramanga, Alaotra-Mangoro, Madagascar	[29]
Lagascatriol (**27**)	322	C_20_H_34_O_3_	*Sideritis serata* Lag. (*Lamiaceae*), aerial parts, Albacete, Southeast of Spain in Sierra de Ben & near Tobarra	[15]
Lagascol (**28**)	306	C_20_H_34_O_2_	*Sideritis serata* Lag. (*Lamiaceae*), aerial parts, Albacete, Southeast of Spain in Sierra de Ben & near Tobarra	[15]
Euphoainoid H (**29**)	300	C_20_H_28_O_2_	*Euphorbia milii* Des Moul., (*Euphorbiaceae*), aerial parts, Baoshan in Yunnan, China	[30]
Candidenodiol (**30**)	318	C_20_H_30_O_3_	*Vellozia candida* Mikan (*Velloziaceae*), whole plant, Corcovado Mountain, Rio de Janeiro, RJ, Brazil	[31]
Anomalusin A (**31**)	318	C_20_H_30_O_3_	*Mallotus anomalus* Meer et Chun, (*Euphorbiaceae*), root, Hainan, China	[32]
Anomalusin B (**32**)	318	C_20_H_30_O_3_	*Mallotus anomalus* Meer et Chun, (*Euphorbiaceae*), root, Hainan, China	[32]
Sagittine A (**33**)	302	C_20_H_30_O_2_	*Sagittaria sagittifolia* L., (*Alismaceae*), herb, Nanning, Guangxi, China	[25]
Sagittine B (**34**)	420	C_25_H_40_O	*Sagittaria sagittifolia* L., (*Alismaceae*), herb, Nanning, Guangxi, China	[25]
			*Sagittaria trifolia* var. *sinensis* (*Sims*) Makino, (*Alismataceae*), herb, Nanning, Guangxi Zhuang Autonomous Region, China	[18]
Sagittine C (**35**)	462	C_27_H_42_O_6_	*Sagittaria sagittifolia* L., (*Alismaceae*), herb, Nanning, Guangxi, China	[25]
Sagittine D (**36**)	462	C_27_H_42_O_6_	*Sagittaria sagittifolia* L., (*Alismaceae*), herb, Nanning, Guangxi, China	[25]
Sagittine E (**37**)	504	C_29_H_44_O_7_	*Sagittaria sagittifolia* L., (*Alismaceae*), herb, Nanning, Guangxi, China	[25]
			*Sagittaria trifolia* var. *sinensis* (*Sims*) Makino, (*Alismataceae*), herb, Nanning, Guangxi Zhuang Autonomous Region, China	[18]
Sagittine F (**38**)	504	C_29_H_44_O_7_	*Sagittaria sagittifolia* L., (*Alismaceae*), herb, Nanning, Guangxi, China	[25]
			*Sagittaria trifolia* var. *sinensis* (*Sims*) Makino, (*Alismataceae*), herb, Nanning, Guangxi Zhuang Autonomous Region, China	[18]
ent-Rosane diterpenoids with Δ^1^(^10^) unsaturation in rings A and B				
(3*R*)-ent-Rosa-1(10),15-dien-3α-ol (**39**)	288	C_20_H_32_O	*Plagiochila deltoidea* Lindenb. (NZ-128) (*Plagiochilaceae*), aerial parts, Sewell Peak, Ohakune, Te Aroha and Haast in New Zealand	[33]
			*Euphorbia fischeriana* Steud., (*Euphorbiaceae*), root, Anhui, China	[34]
(3*R*,15*R*)-ent-15,16-Epoxy-1(10)--rosen-3-ol (**40**)	304	C_20_H_32_O_2_	*Plagiochila deltoidea* Lindenb. (NZ-128) (*Plagiochilaceae*), aerial parts, Sewell Peak, Ohakune, Te Aroha and Haast in New Zealand	[33]
			*Euphorbia ebracteolata* Hayata, (*Euphorbiaceae*), root, Changchun, Jilin, China	[24]
Euphorpekone A (**41**)	302	C_20_H_30_O_2_	*Euphorbia pekinensis* Rupr., (*Euphorbiaceae*), herb, Jinzhong, Shanxi, China	[35]
Euphorpekone B (**42**)	302	C_20_H_30_O_2_	*Euphorbia pekinensis* Rupr., (*Euphorbiaceae*), herb, Jinzhong, Shanxi, China	[35]
Euphominoid J (**43**)	302	C_20_H_30_O_2_	*Euphorbia milii* Des Moul., (*Euphorbiaceae*), aerial parts, Baoshan, Yunnan, China	[36]
5-epi-Euphominoid J (**44**)	302	C_20_H_30_O_2_	*Euphorbia milii* Des Moul., (*Euphorbiaceae*), aerial parts, Baoshan, Yunnan, China	[36]
Euphominoid K (**45**)	318	C_20_H_30_O_3_	*Euphorbia milii* Des Moul., (*Euphorbiaceae*), aerial parts, Baoshan, Yunnan, China	[36]
Euphominoid L (**46**)	318	C_20_H_30_O_3_	*Euphorbia milii* Des Moul., (*Euphorbiaceae*), aerial parts, Baoshan, Yunnan, China	[36]
Δ^5^(^10^),Δ^15^(^16^)-unsaturated ent-rosane diterpenois				
Euphominoid A (**47**)	302	C_20_H_30_O_2_	*Euphorbia milii* Des Moul., (*Euphorbiaceae*), aerial parts, Baoshan, Yunnan, China	[36]
			*Euphorbia milii* Des Moul., (*Euphorbiaceae*), aerial parts, Baoshan in Yunnan, China	[30]
Euphominoid B (**48**)	304	C_20_H_32_O_2_	*Euphorbia milii* Des Moul., (*Euphorbiaceae*), aerial parts, Baoshan, Yunnan, China	[36]
Euphominoid C (**49**)	304	C_20_H_32_O_2_	*Euphorbia milii* Des Moul., (*Euphorbiaceae*), aerial parts, Baoshan, Yunnan, China	[36]
Euphominoid D (**50**)	286	C_20_H_30_O	*Euphorbia milii* Des Moul., (*Euphorbiaceae*), aerial parts, Baoshan, Yunnan, China	[36]
Euphominoid E (**51**)	302	C_20_H_30_O_2_	*Euphorbia milii* Des Moul., (*Euphorbiaceae*), aerial parts, Baoshan, Yunnan, China	[36]
			*Euphorbia neriifolia* L., (*Euphorbiaceae*), stems, Nanning, Guangxi Zhuang Autonomous Region, China	[37]
Euphominoid F (**52**)	302	C_20_H_30_O_2_	*Euphorbia milii* Des Moul., (*Euphorbiaceae*), aerial parts, Baoshan, Yunnan, China	[36]
Euphominoid G (**53**)	316	C_20_H_28_O_3_	*Euphorbia milii* Des Moul., (*Euphorbiaceae*), aerial parts, Baoshan, Yunnan, China	[36]
Euphominoid H (54)	318	C_20_H_30_O_3_	*Euphorbia milii* Des Moul., (*Euphorbiaceae*), aerial parts, Baoshan, Yunnan, China	[36]
Euphominoid I (**55**)	318	C_20_H_30_O_3_	*Euphorbia milii* Des Moul., (*Euphorbiaceae*), aerial parts, Baoshan, Yunnan, China	[36]
Euphomillanol C (**56**)	334	C_20_H_30_O_4_	*Euphorbia milii* Des Moul., (*Euphorbiaceae*), aerial parts, Menglun town of Yunnan, China	[38]
Euphomillanol D (**57**)	322	C_20_H_34_O_3_	*Euphorbia milii* Des Moul., (*Euphorbiaceae*), aerial parts, Menglun town of Yunnan, China	[38]
Euphomillanol E (**58**)	288	C_20_H_32_O	*Euphorbia milii* Des Moul., (*Euphorbiaceae*), aerial parts, Menglun town of Yunnan, China	[38]
Euphoainoid A (**59**)	480	C_30_H_40_O_5_	*Euphorbia milii* Des Moul., (*Euphorbiaceae*), aerial parts, Baoshan in Yunnan, China	[30]
Euphoainoid B (**60**)	450	C_29_H_38_O_4_	*Euphorbia milii* Des Moul., (*Euphorbiaceae*), aerial parts, Baoshan in Yunnan, China	[30]
Euphoainoid C (**61**)	346	C_22_H_34_O_3_	*Euphorbia milii* Des Moul., (*Euphorbiaceae*), aerial parts, Baoshan in Yunnan, China	[30]
Euphoainoid D (**62**)	478	C_30_H_38_O_5_	*Euphorbia milii* Des Moul., (*Euphorbiaceae*), aerial parts, Baoshan in Yunnan, China	[30]
Euphoainoid E (**63**)	448	C_29_H_36_O_4_	*Euphorbia milii* Des Moul., (*Euphorbiaceae*), aerial parts, Baoshan in Yunnan, China	[30]
Euphoainoid F (**64**)	346	C_22_H_34_O_3_	*Euphorbia milii* Des Moul., (*Euphorbiaceae*), aerial parts, Baoshan in Yunnan, China	[30]
Euphoainoid G (**65**)	318	C_20_H_30_O_3_	*Euphorbia milii* Des Moul., (*Euphorbiaceae*), aerial parts, Baoshan in Yunnan, China	[30]
Euphoainoid I (**66**)	332	C_21_H_32_O_3_	*Euphorbia milii* Des Moul., (*Euphorbiaceae*), aerial parts, Baoshan in Yunnan, China	[30]
Euphoainoid J (**67**)	272	C_19_H_28_O	*Euphorbia milii* Des Moul., (*Euphorbiaceae*), aerial parts, Baoshan in Yunnan, China	[30]
Other unsaturated ent-rosane diterpenoids				
Blusamiferoid C (**68**)	318	C_20_H_28_O_3_	*Blumea balsamifera* L. DC., (*Asteraceae*), aerial parts, purchased from Baoding Xiande Chinese Medicine Sales Co., Ltd., Guizhou, China	[39]
Euphorin A (**69**)	302	C_20_H_30_O_2_	*Euphorbia fischeriana* Steud., (*Euphorbiaceae*), root, Anhui, China	[34]
			*Euphorbia ebracteolata* Hayata, (*Euphorbiaceae*), root, Bozhou, Anhui, China	[40]
			*Euphorbia ebracteolata* Hayata, (*Euphorbiaceae*), root, Chuzhou, Anhui, China	[19]
Euphorin B (**70**)	318	C_20_H_30_O_3_	*Euphorbia fischeriana* Steud., (*Euphorbiaceae*), root, Anhui, China	[34]
			*Euphorbia ebracteolata* Hayata, (*Euphorbiaceae*), root, Chuzhou, Anhui, China	[19]
Epoxicorcovadin (**71**)	316	C_20_H_28_O_3_	*Vellozia candida* Mikan (*Velloziaceae*), stem, roots and leaf sheaths, grows on the hillside of Corcovado Mountain and on the Rocky Mountains of the coast of State of Rio de Janeiro	[16]
Epoxivellozin (**72**)	332	C_20_H_28_O_4_	*Vellozia candida* Mikan (*Velloziaceae*), stem, roots and leaf sheaths, grows on the hillside of Corcovado Mountain and on the Rocky Mountains of the coast of State of Rio de Janeiro	[16]
Omphaline A (**73**)	304	C_19_H_28_O_3_	of *Omphalea oppositifolia* (*Willd*.) L.J. Gillespie, (Euphorbiaceae), leaves and twigs, Moramanga, Alaotra-Mangoro, Madagascar	[29]
Aromatic ent-rosane diterpenoids				
Euphorin C (**74**)	314	C_19_H_22_O_4_	*Euphorbia fischeriana* Steud., (*Euphorbiaceae*), root, Anhui, China	[34]
			*Euphorbia ebracteolata* Hayata, (*Euphorbiaceae*), root, Chuzhou, Anhui, China	[19]
Nematocynine (**75**)	286	C_19_H_26_O_2_	*Euphorbia nematocypha* (*Euphorbiaceae*), root, purchased in the Luosiwan pharmacy market, Kunming, China	[41]
Euphoainoid K (**76**)	300	C_20_H_28_O_2_	*Euphorbia milii* Des Moul., (*Euphorbiaceae*), aerial parts, Baoshan in Yunnan, China	[30]
Euphoainoid L (**77**)	300	C_20_H_28_O_2_	*Euphorbia milii* Des Moul., (*Euphorbiaceae*), aerial parts, Baoshan in Yunnan, China	[30]
Modified skeletons ent-rosane diterpenoids				
Euphnerin A (**78**)	320	C_20_H_32_O_3_	*Euphorbia neriifolia* L., (*Euphorbiaceae*), stems, Nanning, Guangxi Zhuang Autonomous Region, China	[37]
Euphnerin B (**79**)	304	C_20_H_32_O_2_	*Euphorbia neriifolia* L., (*Euphorbiaceae*), stems, Nanning, Guangxi Zhuang Autonomous Region, China	[37]
Euphorin D (**80**)	304	C_19_H_28_O_3_	*Euphorbia fischeriana* Steud., (*Euphorbiaceae*), root, Anhui, China	[34]
			*Euphorbia ebracteolata* Hayata, (*Euphorbiaceae*), root, Bozhou, Anhui, China	[40]
			*Euphorbia ebracteolata* Hayata, (*Euphorbiaceae*), root, Chuzhou, Anhui, China	[19]
Fischeria A (**81**)	288	C_19_H_28_O_2_	*Euphorbia fischeriana* Steud., (*Euphorbiaceae*), rhizome, China	[17]
			*Euphorbia fischeriana* Steud., (*Euphorbiaceae*), root, Anhui, China	[34]
			*Euphorbia ebracteolata* Hayata, (*Euphorbiaceae*), root, Bozhou, Anhui, China	[40]
Euphomilone A (**82**)	320	C_20_H_32_O_3_	*Euphorbia milii* Des Moul., (*Euphorbiaceae*), aerial parts, Baoshan, Yunnan, China	[26]
Euphomilone B (**83**)	320	C_20_H_32_O_3_	*Euphorbia milii* Des Moul., (*Euphorbiaceae*), aerial parts, Baoshan, Yunnan, China	[26]
Euphomilone F (**84**)	336	C_20_H_32_O_4_	*Euphorbia milii* Des Moul., (*Euphorbiaceae*), aerial parts, Baoshan in Yunnan, China	[30]
Euphomillanol A (**85**)	318	C_20_H_30_O_3_	*Euphorbia milii* Des Moul., (*Euphorbiaceae*), aerial parts, Menglun town of Yunnan, China	[38]
Euphomillanol B (**86**)	302	C_20_H_30_O_2_	*Euphorbia milii* Des Moul., (*Euphorbiaceae*), aerial parts, Menglun town of Yunnan, China	[38]
Dimeric ent-rosane diterpenoids				
Eupractenoid A (**87**)	616	C_39_H_52_O_6_	*Euphorbia ebracteolata* Hayata, (*Euphorbiaceae*), root, Bozhou, Anhui, China	[42]
Eupractenoid B (**88**)	600	C_39_H_52_O_5_	*Euphorbia ebracteolata* Hayata, (*Euphorbiaceae*), root, Bozhou, Anhui, China	[42]
Bisebracteolasin A (**89**)	664	C_40_H_56_O_8_	*Euphorbia ebracteolata* Hayata, (*Euphorbiaceae*), root, Anhui, China	[43]
Bisebracteolasin B (**90**)	666	C_40_H_58_O_8_	*Euphorbia ebracteolata* Hayata, (*Euphorbiaceae*), root, Anhui, China	[43]

**Table 2.** List of reported rosane diterpenoids (molecular weights, formulae, source and location)

Compound name/class	Molecular weight	Molecular formula	Plant, family, part used and location	Ref.
Lactone-containing rosane diterpenoids				
Rosenonolactone (**91**)	316	C_20_H_28_O_3_	*Trichothecium roseum* (CM1 50,660), fungus, *Hypocreaceae*, cultured, United Kingdom	[44]
			*Trichothecium roseum* (F1064), fungus, *Hypocreaceae*, soil sample, Mt. Jiree, Kyungnam, Korea	[45]
			*Engleromyces goetzii* Henn., cultured fruiting bodies (*Xylariaceae*), Shangri-La, Yunnan Provice, China	[48]
			*Jatropha curcas* L., (*Euphorbiaceae*), branches and leaves, Yuanjiang, Yunnan, China	[47]
7-Deoxyrosenonolactone (**92**)	302	C_20_H_30_O_2_	*Trichothecium roseum* (CM1 50,660), fungus, *Hypocreaceae*, cultured, United Kingdom	[44]
			*Trichothecium roseum* (F1064), fungus, *Hypocreaceae*, soil sample, Mt. Jiree, Kyungnam, Korea	[45]
			*Engleromyces goetzii* Henn., cultured fruiting bodies (*Xylariaceae*), Shangri-La, Yunnan Provice, China	[48]
			*Jatropha curcas* L., (*Euphorbiaceae*), branches and leaves, Yuanjiang, Yunnan, China	[47]
8*S*-Rosenonolactone (**93**)	316	C_20_H_28_O_3_	*Jatropha curcas* L., (*Euphorbiaceae*), branches and leaves, Yuanjiang, Yunnan, China	[47]
11β-Hydroxyrosenonolactone (**94**)	332	C_20_H_28_O_4_	*Trichothecium roseum* (CM1 50,660), fungus, *Hypocreaceae*, cultured, United Kingdom	[44]
Lyrosenonolactone (**95**)	332	C_20_H_28_O_4_	*Lycoris aurea* L'Herit., (*Amaryllidaceae*), whole plant, Kunming, Yunnan, China	[46]
Rosenololactone (**96**)	318	C_20_H_30_O_3_	*Trichothecium roseum* (CM1 50,660), fungus, *Hypocreaceae*, cultured, United Kingdom	[44]
			*Trichothecium roseum* (F1064), fungus, *Hypocreaceae*, soil sample, Mt. Jiree, Kyungnam, Korea	[45]
Rosololactone (**97**)	318	C_20_H_30_O_3_	*Trichothecium roseum* (CM1 50,660), fungus, *Hypocreaceae*, cultured, United Kingdom	[44]
			*Lycoris aurea* L'Herit., (*Amaryllidaceae*), whole plant, Kunming, Yunnan, China	[46]
			*Engleromyces goetzii* Henn., cultured fruiting bodies (*Xylariaceae*), Shangri-La, Yunnan Provice, China	[48]
			*Jatropha curcas* L., (*Euphorbiaceae*), branches and leaves, Yuanjiang, Yunnan, China	[47]
Candidalactone (**98**)	332	C_20_H_28_O_4_	*Vellozia candida* Mikan (*Velloziaceae*), whole plant, Corcovado Mountain, Rio de Janeiro, RJ, Brazil	[31]
(3a1*R*,5a*R*,6a*S*,8*R*,10a*R*)-8,10a-dimethyl-8-vinyl-2,3a1,5a,6,6a,7,8,9,10,10a-decahydro-4*H*-phenanthro[10,1-bc]furan-4-one (**99**)	284	C_19_H_24_O_2_	*Stachys parviflora* Benth., (*Lamiaceae*), whole plant, Abbottabad, Pakistan	[49]
(3a*S*,3a1*R*,5a*R*,6a*S*,8*R*,10a*R*)-3a,8,10a-trimethyl-8-vinyl-2,3,3a,3a1,5a,6,6a,7,8,9,10,10a-dodecahydro-4*H*-phenanthro[10,1-bc]furan-4-one (**100**)	300	C_20_H_28_O_2_	*Stachys parviflora* Benth., (*Lamiaceae*), whole plant, Abbottabad, Pakistan	[50]
Ebractenoid A (**101**)	304	C_19_H_28_O_3_	*Euphorbia ebracteolata* Hayata, (*Euphorbiaceae*), root, purchased from Traditional Chinese Medicinal Materials Trading Center, Bozhou, Anhui, China	[51]
			*Euphorbia ebracteolata* Hayata, (*Euphorbiaceae*), root, Chuzhou, Anhui, China	[19]
Ebractenoid B (**102**)	304	C_19_H_28_O_3_	*Euphorbia ebracteolata* Hayata, (*Euphorbiaceae*), root, purchased from Traditional Chinese Medicinal Materials Trading Center, Bozhou, Anhui, China	[51]
			*Euphorbia ebracteolata* Hayata, (*Euphorbiaceae*), root, Bozhou, Anhui, China	[40]
			*Euphorbia ebracteolata* Hayata, (*Euphorbiaceae*), root, Chuzhou, Anhui, China	[19]
Ebractenoid R (**103**)	286	C_19_H_26_O_2_	*Euphorbia ebracteolata* Hayata, (*Euphorbiaceae*), root, Bozhou, Anhui, China	[40]
Ebractenoid S (**104**)	316	C_20_H_28_O_3_	*Euphorbia ebracteolata* Hayata, (*Euphorbiaceae*), root, Bozhou, Anhui, China	[40]
Ketone-containing rosane diterpenoids				
Hugorosenone (**105**)	302	C_20_H_30_O_2_	*Hugonia casteneifolia* Engl, (*Linaceae*), root bark, Pugu Forest, Tanzania	[52]
			*Hugonia castaneifolia* Engl., (*Linaceae*), root bark, Pugu forest, Tanzania	[53]
18-Hydroxyhugorosenone (**106**)	318	C_20_H_30_O_3_	*Hugonia casteneifolia* Engl, (*Linaceae*), root bark, Pugu Forest, Tanzania	[52]
			*Hugonia castaneifolia* Engl., (*Linaceae*), root bark, Pugu forest, Tanzania	[53]
18-Hydroxy-3-deoxyhugorosenone (**107**)	302	C_20_H_30_O_2_	*Hugonia castaneifolia* Engl., (*Linaceae*), root bark, Pugu forest, Tanzania	[53]
3β,19-Dihydroxy-1(10),15-rosadien-2-one (**108**)	318	C_20_H_30_O_3_	*Euphorbia ebracteolata* Hayata, (*Euphorbiaceae*), roots, Changchun, Jilin, China	[54]
			*Euphorbia ebracteolata* Hayata, (*Euphorbiaceae*), root, purchased from Traditional Chinese Medicinal Materials Trading Center, Bozhou, Anhui, China	[51]
			*Euphorbia nematocypha* (*Euphorbiaceae*), root, purchased in the Luosiwan pharmacy market, Kunming, China	[41]
5,15-Rosadiene-3,11-dione (**109**)	300	C_20_H_28_O_2_	*Tylimanthus renifolius* Hässel et Solari (*Acrobolbaceae*) liverwort, Ushuaia in a Nothofagus pumilio forest, Tierra del Fuego, Argentina	[55]
			Unidentified Argentine liverwort *Anastrophyllum* species, (*Anastrophyllaceae*)	[56]
33-Hydroxy-5,15-rosadien-11-one (**110**)	302	C_20_H_30_O_2_	Unidentified Argentine liverwort *Anastrophyllum* species, (*Anastrophyllaceae*)	[56]
Anomallotusinsin (**111**)	320	C_20_H_32_O_3_	*Mallotus anomalus* Meer et Chun, (*Euphorbiaceae*), root, Hainan, China	[32]
Euphebracteolatin C (**112**)	302	C_20_H_30_O_2_	*Euphorbia ebracteolata* Hayata, (*Euphorbiaceae*), root, Chuzhou, Anhui, China	[19]
Ebractenoid H (**113**)	318	C_20_H_30_O_3_	*Euphorbia ebracteolata* Hayata, (*Euphorbiaceae*), root, purchased from Traditional Chinese Medicinal Materials Trading Center, Bozhou, Anhui, China	[51]
			*Euphorbia ebracteolata* Hayata, (*Euphorbiaceae*), root, Chuzhou, Anhui, China	[19]
Ebractenoid I (**114**)	318	C_20_H_30_O_3_	*Euphorbia ebracteolata* Hayata, (*Euphorbiaceae*), root, purchased from Traditional Chinese Medicinal Materials Trading Center, Bozhou, Anhui, China	[51]
Ebractenoid J (**115**)	300	C_20_H_28_O_2_	*Euphorbia ebracteolata* Hayata, (*Euphorbiaceae*), root, purchased from Traditional Chinese Medicinal Materials Trading Center, Bozhou, Anhui, China	[51]
			*Euphorbia ebracteolata* Hayata, (*Euphorbiaceae*), root, Bozhou, Anhui, China	[40]
Ebractenoid O (**116**)	282	C_20_H_26_O	*Euphorbia ebracteolata* Hayata, (*Euphorbiaceae*), root, Bozhou, Anhui, China	[40]
Ebractenoid P (**117**)	284	C_20_H_28_O	*Euphorbia ebracteolata* Hayata, (*Euphorbiaceae*), root, Bozhou, Anhui, China	[40]
Ebractenoid Q (**118**)	288	C_20_H_28_O	*Euphorbia ebracteolata* Hayata, (*Euphorbiaceae*), root, Bozhou,Anhui, China	[40]
3-Oxo-5,6-epoxyros-15-en-18--acetate (**119**)	360	C_22_H_32_O_4_	*Trichogonia salviaefolia* Gardner (*Asteraceae*), aerial parts, Bahia, Brazil,	[20]
3-Oxorosa-5,15-dien-18-acetate (**120**)	344	C_22_H_32_O_3_	*Trichogonia salviaefolia* Gardner (*Asteraceae*), aerial parts, Bahia, Brazil,	[20]
Rosadiene and ester derivatives				
Rosa-5,15-dien-3α,18-diol (**121**)	304	C_20_H_32_O_2_	*Trichogonia salviaefolia* Gardner (*Asteraceae*), aerial parts, Bahia, Brazil	[20]
18-Hydroxyrosa-5,15-dien--3α-acetate (**122**)	346	C_22_H_34_O_3_	*Trichogonia villosa* Sch.Bip. ex Baker (*Asteraceae*), aerial parts, Bahia, Brazil	[20]
3α-Hydroxyrosa-5,15-dien--18-acetate (**123**)	346	C_22_H_34_O_3_	*Trichogonia villosa* Sch.Bip. ex Baker (*Asteraceae*), aerial parts, Bahia, Brazil	[20]
18-Oxorosa-5,15-dien-3α-acetate (**124**)	344	C_22_H_32_O_3_	*Trichogonia salviaefolia* Gardner (*Asteraceae*), aerial parts, Bahia, Brazil,	[20]
Rosa-1(10),15-dien-18-acid (**125**)	302	C_20_H_30_O_2_	*Trichogonia salviaefolia* Gardner (*Asteraceae*), aerial parts, Bahia, Brazil	[20]
			*Trichogonia villosa* Sch.Bip. ex Baker (*Asteraceae*), aerial parts, Bahia, Brazil	[20]
Rosa-1(10),15-dien-18-acid methyl ether (**126**)	316	C_21_H_32_O_2_	*Trichogonia salviaefolia* Gardner (*Asteraceae*), aerial parts, Bahia, Brazil,	[20]
Hugorosenol (**127**)	304	C_20_H_32_O_2_	*Hugonia castaneifolia* Engl., (*Linaceae*), root bark, Pugu forest, Tanzania	[53]
			*Euphorbia ebracteolata* Hayata, (*Euphorbiaceae*), root, purchased from Traditional Chinese Medicinal Materials Trading Center, Bozhou, Anhui, China	[51]
Hugorosediol (**128**)	304	C_20_H_32_O_2_	*Hugonia casteneifolia* Engl, (*Linaceae*), root bark, Pugu Forest, Tanzania	[52]
Yuexiandajisu F (**129**)	304	C_20_H_32_O_2_	*Euphorbia ebracteolata* Hayata (*Euphorbiaceae*), root, Qingzhou, Shangdong, China,	[57]
			*Euphorbia ebracteolata* Hayata, (*Euphorbiaceae*), root, purchased from Traditional Chinese Medicinal Materials Trading Center, Bozhou, Anhui, China	[51]
			*Euphorbia fischeriana* Steud., (*Euphorbiaceae*), root, Anhui, China	[34]
			*Euphorbia ebracteolata* Hayata, (*Euphorbiaceae*), root, Chuzhou, Anhui, China	[19]
19-Hydroxy-1(10),15-rosadiene (**130**)	288	C_20_H_32_O	*Thyrsanthera suborbicularis* PIERRE ex GAGNEP, (*Euphorbiaceae*), Whole plant, Kandal province, Cambodia	[58]
Engleromycenol (**131**)	288	C_20_H_32_O	*Engleromyces goetzii* Henn., cultured fruiting bodies (*Xylariaceae*), Shangri-La, Yunnan Provice, China	[48]
Ebractenoid C (**132**)	304	C_20_H_32_O_2_	*Euphorbia ebracteolata* Hayata, (*Euphorbiaceae*), root, purchased from Traditional Chinese Medicinal Materials Trading Center, Bozhou, Anhui, China	[51]
			*Euphorbia fischeriana* Steud., (*Euphorbiaceae*), root, Anhui, China	[34]
			*Euphorbia ebracteolata* Hayata, (*Euphorbiaceae*), root, Bozhou, Anhui, China	[40]
Ebractenoid D (**133**)	304	C_20_H_32_O_2_	*Euphorbia ebracteolata* Hayata, (*Euphorbiaceae*), root, purchased from Traditional Chinese Medicinal Materials Trading Center, Bozhou, Anhui, China	[51]
			*Euphorbia ebracteolata* Hayata, (*Euphorbiaceae*), root, Bozhou, Anhui, China	[40]
			*Euphorbia ebracteolata* Hayata, (*Euphorbiaceae*), root, Chuzhou, Anhui, China	[19]
Ebractenoid E (**134**)	304	C_20_H_32_O_2_	*Euphorbia ebracteolata* Hayata, (*Euphorbiaceae*), root, purchased from Traditional Chinese Medicinal Materials Trading Center, Bozhou, Anhui, China	[51]
Engleromycenolic acid A (**135**)	318	C_20_H_30_O_3_	*Engleromyces goetzii* Henn., cultured fruiting bodies (*Xylariaceae*), Shangri-La, Yunnan Provice, China	[48]
Engleromycenolic acid B (**136**)	318	C_20_H_30_O_3_	*Engleromyces goetzii* Henn., cultured fruiting bodies (*Xylariaceae*), Shangri-La, Yunnan Provice, China	[48]
Stachysrosane (**137**)*	302	C_20_H_30_O_2_	*Stachys parviflora* Benth., (*Lamiaceae*), whole plant, Abbottabad, Pakistan	[59]
			*Phlomidoschema parviflorum* (*Benth*.) Vved. (Basionym: *Stachys parviflora Benth*.), (*Lamiaceae*), whole plant, Abbottabad, Pakistan	[60]
Stachysrosane (138)*	286	C_19_H_26_O_2_	*Stachys parviflora* Benth., (*Lamiaceae*), whole plant, Abbottabad, Pakistan	[59]
			*Phlomidoschema parviflorum* (*Benth*.) Vved. (Basionym: *Stachys parviflora Benth*.), (Lamiaceae), whole plant, Abbottabad, Pakistan	[60]
Aromatic rosane diterpenoids				
Euphebracteolatin A (**139**)	286	C_19_H_26_O_2_	*Euphorbia ebracteolata* Hayata, (*Euphorbiaceae*), root, Changchun, Jilin, China,	[24]
			*Euphorbia nematocypha* (*Euphorbiaceae*), root, purchased in the Luosiwan pharmacy market, Kunming, China	[41]
			*Euphorbia ebracteolata* Hayata, (*Euphorbiaceae*), root, Chuzhou, Anhui, China	[19]
Euphebracteolatin D (**140**)	286	C_19_H_26_O_2_	*Euphorbia ebracteolata* Hayata, (*Euphorbiaceae*), root, Chuzhou, Anhui, China	[19]
Euphebracteolatin E (**141**)	286	C_19_H_26_O_2_	*Euphorbia ebracteolata* Hayata, (*Euphorbiaceae*), root, Chuzhou, Anhui, China	[19]
Ebractenoid F (**142**)	286	C_19_H_26_O_2_	*Euphorbia ebracteolata* Hayata, (*Euphorbiaceae*), root, purchased from Traditional Chinese Medicinal Materials Trading Center, Bozhou, Anhui, China	[51]
			*Euphorbia fischeriana* Steud., (*Euphorbiaceae*), root, Anhui, China	[34]
			*Euphorbia fischeriana* Steud., (*Euphorbiaceae*), root, Yunnan, China	[61]
Ebractenoid G (**143**)	302	C_19_H_26_O_3_	*Euphorbia ebracteolata* Hayata, (*Euphorbiaceae*), root, purchased from Traditional Chinese Medicinal Materials Trading Center, Bozhou, Anhui, China	[51]
Ebraphenol A (**144**)	268	C_19_H_24_O	*Euphorbia ebracteolata* Hayata, (*Euphorbiaceae*), root, Bozhou, Anhui, China	[62]
Ebraphenol B (**145**)	300	C_20_H_28_O_2_-	*Euphorbia ebracteolata* Hayata, (*Euphorbiaceae*), root, Bozhou, Anhui, China	[62]
			*Euphorbia ebracteolata* Hayata, (*Euphorbiaceae*), root, Bozhou, Anhui, China	[63]
Ebraphenol C (**146**)	270	C_19_H_26_O	*Euphorbia ebracteolata* Hayata, (*Euphorbiaceae*), root, Bozhou, Anhui, China	[62]
Ebraphenol D (**147**)	286	C_19_H_26_O_2_	*Euphorbia ebracteolata* Hayata, (*Euphorbiaceae*), root, Bozhou, Anhui, China	[62]
Ebraphenol E (**148**)	300	C_20_H_28_O_2_	*Euphorbia ebracteolata* Hayata, (*Euphorbiaceae*), root, Bozhou, Anhui, China	[63]
Ebracteolatas D (**149**)	300	C_19_H_24_O_3_	*Euphorbia fischeriana* Steud., (*Euphorbiaceae*), root, Yunnan, China	[61]
Ebralactone A (**150**)	296	C_20_H_24_O_2_	*Euphorbia ebracteolata* Hayata, (*Euphorbiaceae*), root, Bozhou, Anhui, China	[62]
Dimeric rosane diterpenoids				
Yuexiandajisu D (**151**)	568	C_38_H_48_O_4_	*Euphorbia ebracteolata* Hayata, (*Euphorbiaceae*), roots, Beijing Chinese Medicinal Herbs, China	[64]
			*Euphorbia ebracteolata* Hayata, (*Euphorbiaceae*), root, Changchun, Jilin, China	[24]
			*Euphorbia nematocypha* (*Euphorbiaceae*), root, purchased in the Luosiwan pharmacy market, Kunming, China	[41]

*Compounds have the same name with different structures

## Ent-rosane diterpenoids

In this study, 90 ent-rosane diterpenoids were discussed and categorized into six major classes based on the unsaturation pattern of their skeletons (Table 1).

### Saturated ent-rosane diterpenoids

This group comprises hydrogenated or polyoxygenated ent-rosanes bearing hydroxyl, acetoxy, carbonyl, or carboxyl groups but lacking olefinic bonds. Common compounds include ent-rimuen-5-ol, ent-rimuen-3α,5β-diol, ent-rimuen-5,18-diol, and their acetate/tiglate derivatives, which were separated from *Trichogonia salviaefolia* and *Trichogonia villosa* MeOH extract by TLC impregnated with AgNO_3_ and SiO_2_ CC and identified using MS and NMR tools [20]. Compounds **7** and **8** were isolated from the root bark of *Maytenus cuzcoina* by Sephadex LH-20, SiO_2_ (CH_2_Cl_2_:Et_2_O of increasing polarity), and preparative HPTLC developed with n-hexane: Et_2_O (4:6). Their stereo-structures were elucidated using spectroscopic analysis, computational data, and the Riguera ester procedure (Figure 4) [21].

Compound **10** was isolated from the herb of *Sagittaria pygmaea* by silica-gel chromatography [23]. Compound **11** was isolated from the roots of *Euphorbia ebracteolata* [24]. Compounds **12** and 13 were isolated from *Sagittaria sagittifolia* and *S. trifolia var. sinensis* [18,25]. Santos *et al*. [22] isolated compounds **9** and **15**-**17** from the roots and heartwood of *E. barbatum* together with other hydroxylated analogues.

### Ent-Rosane diterpenoids with δ^4^(^5^) unsaturation in rings A and B

This class includes ent-rosane diterpenoids characterized by a double bond between C-5 and C-6. Most of these compounds were isolated from the *Lamiaceae, Euphorbiaceae*, and *Alismataceae* families. For example, compounds **18** and **19** were identified from *Vellozia candida* (*Velloziaceae*) and *Sideritis serata* (*Lamiaceae*) by chromatographic separation of CHCl_3_ extracts on Al_2_O_3_ and SiO_2_ columns [27]. Compounds **20-22** were obtained from the aerial parts of *C. niveus* [28] (Figure 5). Omphalines B-E (23-26) were isolated from the stems of *Omphalea oppositifolia* [29]. These compounds retain the Δ^5^,^15^ diene skeleton but differ in the number and orientation of hydroxyl groups [29]. Compounds **27** and **28** are oxygenated Δ^5^,^15^-dienes isolated from *Sideritis serata* aerial parts [15]. Compound **29** was isolated from the aerial parts of *E. milii* [30], retaining the Δ^5^,^15^ pattern and possessing an oxygen bridge forming a lactone or enone system across C-3 and C-1 [30]. Candidenodiol (**30**) was obtained from the leaves of *V. candida* by Valente et al. [31]. Compounds **31** and **32** were isolated from *Mallotus anomalus* roots [32]. Compounds **33**-**38** are Δ^5^,^15^-unsaturated diterpenes isolated from the rhizomes of *Sagittaria sagittifolia* and *S. trifolia var. sinensis* [18,25].

### Ent-Rosane diterpenoids with Δ^1^(^10^) unsaturation in rings A and B

This structural subclass of ent-rosane diterpenoids is characterized by a double bond at C-1(10) in ring A. Members of this group have mainly been isolated from species belonging to the *Plagiochilaceae* and *Euphorbiaceae* families.

Chromatographic separation of the ether extract of *Plagiochila deltoidea* aerial parts collected in New Zealand using SiO_2_ CC (n-hexane/EtOAc)/ HPLC (n-hexane/EtOAc 9:1) afforded compounds **39** and **40** (Figure 6) [33]. Later, they were isolated from *E. fischeriana* and *E. ebracteolata* [24,34]. Two new ent-rosane diterpenoids, **41** and **42**, containing α,β-unsaturated ketone moieties, were isolated from *Euphorbia pekinensis* Rupr. by SiO_2_ and Sephadex LH-20 CC and elucidated based on NMR, HRMS, X-ray diffraction analysis, and the CD method [35]. Four further Δ^1^(^10^)-unsaturated ent-rosanes, **43**-**46**, were obtained from the aerial parts of *E. milii* collected in Baoshan, Yunnan Province (China) [36].

### Δ5(^10^), Δ^15^(^16^)-Unsaturated ent-rosane (Rosen-1(10),15-diene) diterpenoids

Compounds **47-54**, isolated from the aerial parts of *E. milii* [30,36], represent oxygenated tricyclic ent-rosane diterpenes with variable oxidation at C-3, C-6, C-18, and C-19, displaying Δ^5^,^10^ and Δ^15^,^16^-unsaturation. Their structures were elucidated by extensive NMR, chemical methods, experimental and calculated electronic circular dichroism (ECD), and confirmed by single-crystal X-ray diffraction for euphominoid A, establishing the ent-configuration [30,36]. Further phytochemical investigation of *E. milii* from Menglun (*Yunnan*) led to the identification of compounds 56-58 [38]. Compound **56** features a 1-methyl-6-oxabicyclo[3.2.1]oct-2-ene motif in ring A, which was assigned by NMR, X-ray, and ECD analyses [38] (Figure 7). A study by Peng *et al*. [30] reported compounds **59**-**67** from *Euphorbia milii*, which incorporate aromatic acyl substituents, tetrahydrofuran rings, and 18-nor derivatives.

### Other unsaturated ent-rosane diterpenoids

This group features diverse double-bond positions in rings A, B, and C. For example, compound 68 was obtained from the aerial parts of *Blumea balsamifera* (*Asteraceae*) [39], which retains the ent-rosane 15-ene core with additional double bonds at C-1-C10 and C-8-C14. Compounds **69** and **70** were isolated from the roots of *E. fischeriana*, collected in Anhui, China [34] and later identified in *E. ebracteolata* Hayata from Bozhou and Chuzhou [19,40]. Compounds **71** and **72** were reported from the stem, roots, and leaf sheaths of *Vellozia candida* Mikan (*Velloziaceae*), growing on the hillsides of Corcovado Mountain and the rocky coastal regions of Rio de Janeiro [16]. They possess OH groups at C-7 and C-12, and an epoxide bridge between C-5 and C-10. Compound **73** was isolated from the leaves and twigs of *Omphalea oppositifolia*, collected in Moramanga, Alaotra-Mangoro, Madagascar [29]. This compound is a 19-nor-ent-rosane derivative, lacking the terminal C-20 methyl group, with OH at C-2 and C-6 and Δ^4^, Δ^15^ double bonds.

### Aromatic, modified skeletons, and dimeric ent-rosane diterpenoids

These *compounds* are characterized by aromatic rings within their tetracyclic carbon skeletons, resulting from the oxidative dehydrogenation of the ent-rosane framework, which produces conjugated systems (Figure 8).

Compound **74** was isolated from the roots of *E. fischeriana* collected in Anhui, China. It possesses an aromatic A-ring, bearing oxygenated substituents. Its structure was elucidated by NMR and X-ray analyses [34]. Compound **75** was obtained from the roots of *E. nematocypha* and elucidated by NMR, HRSM, ECD, and optical rotation analyses [41]. Two additional aromatic ent-rosane diterpenes, compounds **76** and **77**, were reported from the aerial parts of *E. milii* [30].

Some ent-rosane diterpenoids exhibit structural modifications or rearrangements of the parent tetracyclic skeleton through oxidation, migration of double bonds, and partial alteration of the ring system. Compounds **78** and **79**, isolated from the stems of *E. neriifolia* L. collected in Guangxi, China, possess oxidatively modified ent-rosane backbones [37]. Compound **80**, obtained from the roots of *E. fischeriana* and *E. ebracteolata*, displays a partially rearranged and oxygenated ent-rosane nucleus [19,34,40]. Similarly, compound **81** was isolated from *E. fischeriana* using SiO_2_ CC and elucidated by NMR and X-ray [17]. Compounds 82-84 were obtained from the aerial parts of *E. milii* in Yunnan, China [26,30]. Compound **84** is a rare ent-rosane-type diterpenoid with a 5/7/6 skeleton. Likewise, **85** and **86**, also isolated from *E. milii*, are unprecedented 7/7/6-fused tricyclic 5,10-seco-*ent*-RDs and possess a unique 11-oxabicyclo[4.4.1]undeca-1(10),5-diene moiety [38].

Compounds **87** and **88** were isolated from the roots of *E. ebracteolata* collected in Bozhou, Anhui Province, China. Both possess dimeric frameworks linked through oxygen bridges between abietane lactone and nor-rosane units [42]. Likewise, compounds **89** and **90** are rare ent-abietane-rosane diterpenoid heterodimers with oxygen-bridged structures also obtained from the roots of *E. ebracteolata* [43].

## Rosane diterpenoids

In this study, 61 rosane diterpenoids were listed. Structurally, these diterpenoids possess notable variations in their skeletons, including lactones, aromatic analogs, simple hydroxyl or keto derivatives, and even dimeric skeletons, highlighting the biosynthetic versatility of this class (Table 2).

### Lactone-containing rosane diterpenoids

Rosenonolactone-type compounds were the first to be described, including **91**, **92**, **94**, and **97** [44,45]. These lactone metabolites were separated from plants (*Lycoris aurea* and *Jatropha curcas*) and fungi (*Trichothecium roseum*) [46,47] (Figure 9).

### Ketone-containing rosane diterpenoids

Several keto derivatives have been identified (Figure 10). These include different *Hugonia* metabolites such as **105-107** [52,53] and108 [54].

### Rosadiene and ester derivatives

*Trichogonia* species yielded rosadiene-type analogs, such as **121** and its acetate derivatives; **122**, **123**, and their ester-modified derivatives (Figure 11) [20]. Additional hydroxylated derivatives (e.g., **127**-**129** and **136**-**138**) illustrate modifications of the rosadiene scaffold.

### Aromatic and dimeric rosane diterpenoids

Several unusual rosane diterpenoids feature aromatic A-rings or rearranged skeletons. For example, **139**-**141** possess an aromatic A-ring system [19], while **144**-**148** and **150** [62] are characterized by additional aromaticity or rare lactonic modifications [61,62]. A dimeric rosane derivative **151**, was reported from *Euphorbia ebracteolate* (Figure 12) [64].

## Biological activities

Rosanoid diterpenoids exhibit diverse bioactivities, including anti-inflammatory, cytotoxic, enzyme inhibitory, antimicrobial, and metabolic modulatory capacities. These bioactivities are directly linked to their structural variations. Their main biological properties are outlined, with an emphasis on structure-activity relationships (SAR) and, when available, mechanistic insights.

### Anti-inflammatory activity

Several studies have examined the anti-inflammatory properties of rosane-type diterpenoids. de las Heras *et al.* [65] reported that compound **27** remarkably prohibited COX-1- and 5-LOX-mediated release of Prostaglandin E2 (PGE_2_) and leukotriene C4, respectively, with a COX-2/COX-1 selectivity ratio of 3.15, indicating selectivity toward COX-1. Compound **68** demonstrated anti-inflammatory activity by significantly suppressing TNF-α secretion in LPS-stimulated macrophages without inducing cytotoxicity and while having no effect on IL-6 and NO [39]. Compounds **78** and **79** inhibited NO production in LPS-induced murine microglial BV-2 cells. Compound **78** showed an IC_50_ of 22.4 μmol L^-1^, compared to 2-methyl-2-thiopseudourea sulfate (SMT) (IC_50_ = 2.0 μmol L^-1^) [37].

Compounds **101**-**102**, **108**, **115**, **127**, **129**, **132**-**134**, **142**, and **143**, isolated from *E. ebracteolata* roots, inhibited LPS-stimulated NO production in RAW 264.7 macrophages with IC_50_ values ranging from 1.02 to 7.50 μmol L^-1^, compared to indomethacin (IC_50_ = 16.67 μmol L^-1^) and hydrocortisone (IC_50_ = 54.23 μmol L^-1^). Among them, **108** demonstrated more powerful NO inhibition (IC_50_ = 1.02 μmol L^-1^) than indomethacin (IC_50_ = 16.67 μmol L^-1^). Additionally, compounds **115** (IC_50_ = 2.76 μmol L^-1^), **142** (IC_50_ = 3.33 μmol L^-1^), and **143** (IC_50_ = 2.44 μmol L^-1^) displayed marked inhibitory effects [51].

Mechanistic study by Chun et al. revealed that **142** remarkably inhibited NO production (IC_50_ = 2.39 μg mL^-1^) and NF-κB activity (IC_50_ = 4.01 μg mL^-1^) in LPS-stimulated RAW 264.7 macrophages. This compound markedly suppressed iNOS mRNA and protein expression, prevented IκB-α phosphorylation and degradation, and inhibited nuclear translocation of NF-κB p65 and p50 subunits. Additionally, it downregulated IL-6 and IL-1β expression and inhibited phosphorylation of JNK, ERK1/2, IKKα/β, and Akt. Thus, **142** exhibited anti-inflammatory efficacy by inhibition of the NF-κB/MAPK/ PI3K/Akt signaling cascades [66]. Compound **130** exhibited potent inhibitory activity against NO production (IC_50_ = 2.91 mg μL^-1^) in LPS-induced RAW264.7 cells. It (doses 10 and 20 mg mL^-1^) suppressed iNOS mRNA expression [58].

### Anti-osteoclastogenic activity

Compounds **60**-**65**, characterized by aromatic ester substitutions, showed potent inhibition of RANKL-induced osteoclastogenesis (IC_50_s = 4.6 to 9.8 μmol L^-1^), compared to alendronate sodium (IC_50_ = 4.4 μmol L^-1^), with compound **62** being the most active (IC_50_ 4.6 = μmol L^-1^) [30].

### Anti-adipogenic and lipid-lowering activity

Compounds **56**-**58** demonstrated potent antiadipogenic capacity and reduced triglyceride levels in 3T3-L1 preadipocyte differentiation assays (EC_50_s = 3.92 to 18.30 μmol L^-1^). Compound **57** was the most potent compound, revealing that the C-15 and C-16 hydroxyl groups play a crucial role in enhancing antiadipogenic activity [38].

### Antiviral activity

Compounds **47**-**49** and **43** showed potential to inhibit EBV lytic DNA replication in P3HR-1 cells (EC_50_s ranging from 5.4 to 29.1 μmol L^-1^), whereas compound **48** showed the strongest inhibition (EC_50_ = 5.4 μM), comparable to (+)-rutamarin (EC_50_ = 5.4 μmol L^-1^). It was found that compounds with a 5,10-double bond exhibited more potent inhibitory activity than those with α,β-unsaturated carbonyl groups against EBV lytic replication [36].

### Anticancer and cytotoxic activities

Several investigations have reported the cytotoxic potential of rosane diterpenoids against various tumour cell lines. Compounds **20** and **21** were evaluated for their cytotoxic activity using sulforhodamine B (SRB) assay *in vitro* against human cancer cell lines: U251 (human glioblastoma), PC-3 (human prostatic adenocarcinoma), K562 (human chronic myelogenous leukemia), HCT-15 (human colo-rectal adenocarcinoma), and MCF-7 (human mammary adenocarcinoma). Compound **21** inhibited 91.05 % of PC-3 (prostate cancer), 83.46 % of HCT-15 (colon cancer) and 73.16 % of MCF-7 (breast cancer) cells, with IC_50_s = 34.76, 41.93, and 77.08 μmol L^-1^, respectively, whereas **20** produced 77.68, 52.11, and 49.17 % inhibition of PC-3, HCT-15, and MCF-7. Both compounds interacted with NF-κB and STAT-3, suggesting that ent-rosane diterpenes, particularly compound **21**, have promising anticancer potential through inhibition of NF-κB and STAT-3 signalling [28].

Ribosomal S6 kinase (RSK) is a key regulator of tumour cell survival and growth. Molecular docking revealed that compound **75** exhibited strong binding affinity for RSK with a binding energy of -36.1 kJ mol^-1^, compared to the reference LJH685 (-33.3 kJ mol^-1^) by forming three hydrogen bonds with Asp211, Lys100, and Asp148. Also, it showed cytotoxic activity against HCC 1806, CT26, and HeLa cells (IC_50_s = 16.96, 52.04 and 52.70 μmol L^-1^, respectively) [41].

Compound **89** displayed potent activity against HL-60, SMMC-7721, and MCF-7 (IC_50_s = 2.61, 4.08, and 8.17 μmol L^-1^, respectively), while **90** was moderately active (IC_50_s = 10.64 to 16.05 μmol L^-1^) [43]. Meanwhile, **105**, separated from *Hugonia castaneifolia*, exhibited cytotoxicity in the brine shrimp lethality bioassay [52]. A study by Ding *et al.* [19] stated that **112** displayed selective cytotoxic capacities against HepG2 cells (IC_50_ = 14.29 μmol L^-1^) compared to cisplatin (IC_50_ = 7.04 μmol L^-1^), while **140** and **141** were moderately active (IC_50_ = 23.69 and 40.85 μmol L^-1^). Interestingly, the compounds with an α,β-unsaturated ketone moiety were more potent than their aromatic analogs, indicating that the conjugated enone functionality played a significant role in cytotoxicity. The new rosane, **149**, obtained from *E. fischeriana*, demonstrated moderate cytotoxic efficacy against A549 lung carcinoma cells (IC_50_ = 22.03 μmol L^-1^). Annexin V staining showed that this compound induced marked apoptosis [61]. Fu *et al.* [64] revealed that the 18-nor-rosane-type dimeric diterpenoid **151** from *Euphorbia ebracteolata*, exhibited marked cytotoxicity toward HCT-8 colon carcinoma and Bel-7402 hepatocellular carcinoma cells (IC_50_s = 2.66 and 3.76 μmol L^-1^, respectively), compared to adriamycin (IC_50_s = 0.21 and 0.48 μmol L^-1^, respectively), while it showed weak effecttiveness against KB, A549, and BGC-823 cell lines, suggesting the dimerization and the 2,3-dihydro-1,4-benzodioxine bridge might enhance the selectivity toward specific tumour targets.

### Anti-Glucosidase and anti-tuberculosis activity

Compound **87** showed moderate α-glucosidase inhibition (IC_50_ = 7.94 μmol L^-1^), while 88 inhibited *Mycobacterium tuberculosis* GlmU acetyltransferase (IC_50_ = 41.85 μmol L^-1^), a key enzyme involved in bacterial cell wall biosynthesis and a promising target for tuberculosis therapy [42].

### Antibacterial activity

These diterpenoids were assessed for their antimicrobial and antimycobacterial activities. Liu *et al.* [23] reported that **10** exhibited mild antibacterial activity against *Streptococcus mutans* and *Actinomyces viscosus* (MIC = 125.0 μg mL^-1^). Compound **13** showed weak inhibition against *S. mutans* and *A. naeslundii* (MIC = 62.5 μg mL^-1^) [18]. Compound **37** was active against *Actinomyces naeslundiis* ATCC 12104 (MIC = 62.5 μg mL^-1^) [25]. Compounds **105**-**107** exhibited antifungal activity against *Cladosporium cucumericum* (inhibition zone diameters 38.47, 63.59 and 12.56 mm^2^, respectively), with **107** demonstrating the highest activity (inhibition zone 12.56 mm^2^) [53]. Yu *et al.* [40] reported that **118** showed moderate inhibition of *Mycobacterium tuberculosis* H_37_Rv (MIC = 18 μg mL^-1^). Compound **118** moderately inhibited GlmU enzyme (IC_50_ = 12.5 μg mL^-1^). GlmU, a bifunctional N-acetylglucosamine-1-phosphate uridyltransferase/acetyltransferase enzyme essential for mycobacterial cell wall biosynthesis [40].

### Enzyme inhibitory and other activities

Some rosane-type diterpenoids demonstrate enzyme-inhibitory properties. For example, compounds **91**, **92** and **97**, isolated from *Trichothecium roseum*, were found to inhibit cholesteryl ester transfer protein (CETP) *in vitro* (IC_50_s = 31, 65 and 60 μg mL^-1^, respectively) [45]. The aromatic derivatives **144-147** and **150**, isolated from the roots of *Euphorbia ebracteolata*, demonstrated notable pancreatic lipase inhibition, a key enzyme involved in dietary fat absorption. Among them, **144** was the most potent (IC_50_ 1= 1.0 μg mL; Ki = 1.8 μg mL^-1^), compared to lovastatin (IC_50_ = 0.24 μmol L^-1^) [62]. Additionally, **148** showed a moderate lipase inhibitory effect (IC_50_ = 12.5 μmol L^-1^) [63]. Besides, **135** obtained from *Engleromyces goetzii*, significantly inhibited CETP activity (IC_50_ 7.55 μmol L^-1^) [48]. Compound **105** showed larvicidal activity against *Anopheles gambiae* mosquito larvae (LC_50_s = 0.3028, 0.0674 and 0.0582 mg mL^-1^ at 24, 48 and 72 h exposure time, respectively) [53]. From *Stachys parviflora*, **137** and **138** demonstrated pronounced antidiarrheal effects in castor oil-induced diarrhoea models in mice [59]. Also, **137** and **138** display sedative and skeletal muscle relaxant properties in behavioural assays. Docking studies revealed their potential interaction with the GABA-A receptor [60].

## Critical perspective on biological activities

Overall, the reported bioactivities of rosanoid diterpenoids are mainly anti-inflammatory and cytotoxic/ /anticancer, with additional studies supporting enzyme inhibition, antimicrobial, antimycobacterial, antiviral, and metabolic modulatory (antiadipogenic/lipid-lowering and anti-osteoclastogenic) effects. In many cases, the activity was linked to specific structural features, including α,β-unsaturated carbonyl motifs, aromatic ester substitutions, and oxygenation patterns that affected potency across assays. Mechanistic support is available for selected examples, which strengthens the biological relevance of some lead structures.

Rosane-type diterpenoids exhibited promising anti-inflammatory effects in various experimental models, particularly by inhibiting NO production in LPS-stimulated macrophages and suppressing COX/LOX-related inflammatory mediators. Notably, compounds **101-102**, **108**, **115**, **127**, **129**, **132**-**134**, **142** and **143** were the most active, showing strong NO inhibition with IC_50_ values of 1.02 to 7.50 μmol L^-1^. Additionally, the compound **142**’s effect was linked to NF-κB/MAPK/PI3K/Akt signalling pathways. In the anti-osteoclastogenic assay, compounds **60**-**65** with aromatic ester substitutions markedly inhibited RANKL-induced osteoclastogenesis (IC_50_ = 4.6 to 9.8 μmol L^-1^), supporting aromatic ester-bearing rosanoids could be promising for further optimization in bone-resorption-related models. Similarly, compounds **56**-**58** lowered accumulation of triglycerides in 3T3-L1 cells (EC_50_ = 3.92 to 18.30 μmol L^-1^), suggesting that C-15 and C-16 hydroxyls play a crucial role in enhancing antiadipogenic activity. In antiviral assays, compounds **47**-**49** and **43** suppressed EBV lytic DNA replication, demonstrating that the analogs with a 5,10-double bond were more potent than those with α,β-unsaturated carbonyl groups, suggesting that unsaturation can affect antiviral activity. In the cytotoxic and anticancer activities, the reported findings revealed that α,β-unsaturated ketone-containing rosanoids were more potent than aromatic analogs. Therefore, this underscores the need to interpret the above findings with deep mechanistic studies to confirm these conclusions.

## Limitations of the reported studies on rosanoid diterpenoids

Most data arise from *in vitro* studies, which are usually carried out on a limited number of cell lines, microbes, or enzyme assays, with a narrow concentration range, and sometimes without direct comparison with reference drugs. Thus, it is difficult to assess the actual effectiveness, selectivity, and relevance of the described effects. Several studies have employed short-term models and single-cell protocols to investigate the anti-inflammatory, anti-osteoclastogenic, and anti-adipogenic/lipid-modulating effects. In many cases, the inhibition of mediators such as NO, cytokines, or RANKL-mediated pathways has been established; however, there is little or no detailed mechanistic investigation (*e.g.*, at the protein and gene expression levels). Cytotoxic and anticancer effects are commonly observed with certain rosanoid diterpenoids, demonstrating IC_50_ values in the micromolar range. However, these findings typically arise from assays on a limited panel of cancer cell lines, with minimal comparison of toxicity towards healthy cells. In addition, there is a lack of *in vivo* studies, pharmacokinetic, and comprehensive toxicity investigations. The inconsistency in experimental designs across studies further complicates comparisons of outcomes. The current data are useful for identifying potential rosiglitazone frameworks; however, they are not yet reliable enough to support robust conclusions regarding their therapeutic applications or feasibility for drug development.

## Conclusion and future research directions

Rosanoid diterpenoids are a structurally distinct but very small subgroup of diterpenes, which include ent-rosane and rosane derivatives, distinguished by a 6/6/6 tricyclic scaffold and a variety of oxidation patterns, ring modifications, and dimeric frameworks. This review summarizes the reported metabolites from liverworts, fungi, and higher plants, particularly from the family Euphorbiaceae plants. These compounds were assessed for multiple biological activities, including anti-inflammatory, anti-osteoclastogenic, anti-adipogenic, lipid-modulating, cytotoxic, antimicrobial, enzyme-inhibitory, and neuroactive effects. Accordingly, rosanoid diterpenoids may be considered promising scaffolds for further assessment as drug-like leads. Therefore, future research should primarily focus on expanding the sources of rosanoid diterpenoids by investigating understudied species and utilizing advanced analytical and metabolomics techniques; investigating their structure-activity relationships through standardized *in vitro* assays and semi-synthetic modification of key scaffolds; and conducting *in vivo* experiments to evaluate their pharmacokinetics, toxicity, and efficacy in relevant disease models. In parallel, integrating ADMET/DMPK profiling for the most active scaffolds would strengthen prioritization and reduce the risk of overinterpreting isolated assay results. In addition, research aimed at understanding molecular mechanisms will play a pivotal role in ascertaining the relevance of these compounds’ therapeutic potential.
